# Modelling Structural Material Damage Using the Cohesive Zone Approach Under Operational Conditions

**DOI:** 10.3390/ma18174039

**Published:** 2025-08-28

**Authors:** Vladislav Kozák, Jiří Vala, Anna Derevianko

**Affiliations:** Institute of Mathematics and Descriptive Geometry, Faculty of Civil Engineering, Brno University of Technology, 613 00 Brno, Czech Republic; vladislav.kozak@vut.cz (V.K.); anna.derevianko@vut.cz (A.D.)

**Keywords:** structural materials, cohesive zone approach, extended finite element method, 62.20.mt, 46.50.+a, 02.70.Dh, 02.70.Bf, 46.35.+z, 02.30.Jr

## Abstract

This article is devoted to the prediction of the service life of selected structural materials under simulated operating conditions. Special attention is paid to the so-called representative volume element, which characterizes the damage behaviour, since it includes a critical number of microdefects. The overall damage prediction is based on the energy approach, and the development of damage comes from the traction separation laws; the shape of the damage varies for different materials. The calculations were performed using the extended finite element method (XFEM), where several minor modifications were made. This method has been successfully used in many areas of engineering sciences for research, simulation, and prediction of the behaviour of structures. XFEM reformulates the continuous boundary and initial value problems into similar variational forms instead of using the classical forms of differential equations. The simulation of fracture and damage phenomena is presented for two different materials: austenitic steel with a pronounced grain structure under creep (viscous) loading conditions and cement pasta reinforced with metal fibres under conditions of predominantly static loading.

## 1. Introduction

Fracturing in structural materials, particularly those employed in civil, mechanical, and aerospace engineering, remains a critical concern due to the catastrophic consequences of crack initiation and propagation under operational loads. These materials are frequently subjected to complex service conditions involving mechanical loading, temperature variations, and environmental exposure, all of which contribute to the degradation of structural integrity over time. Classical linear elastic fracture mechanics (LEFM), established through pioneering works by Griffith, Barenblatt, and Dugdale [1,2], offers essential insights into brittle fracture behaviour. However, LEFM’s limitations become apparent when addressing quasi-brittle or ductile materials, as it neglects the nonlinear phenomena occurring in the fracture process zone (FPZ). These shortcomings necessitate more sophisticated approaches that can capture the progressive nature of damage evolution.

To overcome these limitations, cohesive zone models (CZMs) and the extended finite element method (XFEM) have emerged as powerful alternatives. CZMs simulate the nonlinear traction–separation response in the FPZ, accommodating phenomena such as microcracking, fibre-matrix debonding, and interfacial failure [3,4]. In particular, CZMs enable the integration of material-specific softening behaviours through experimentally derived traction–separation laws, enhancing predictive capabilities. Complementing CZMs, XFEM extends classical finite element formulations by introducing enrichment functions that model discontinuities such as cracks without requirements to mesh updates [5,6]. This method is particularly well-suited for simulating arbitrary crack paths, branching and interactions in complex material systems. It is worth noting that XFEM, as introduced here for simplicity, can represent a larger class of similar FEM-based approaches—namely, GFEM (generalized FEM), PUFEM (partition-of-unity FEM), various upgrades of the original XFEM, etc.; for more details, see [7,8].

Recent research efforts have increasingly combined CZM and XFEM to leverage their respective strengths. This hybrid strategy facilitates efficient modelling of fracture in fibre-reinforced composites, ceramics, and other heterogeneous materials. Notably, works by Kozák and Vala [9,10] have demonstrated the application of these techniques to simulate crack growth under thermal and mechanical loading, incorporating microstructural details via representative volume elements. This study builds upon these developments by presenting an integrated numerical framework for damage prediction in structural materials subjected to operational conditions. The methodology incorporates elastic–plastic constitutive laws, time-dependent creep effects, and cohesive zone damage mechanics, embedded within XFEM-enhanced finite element simulations. Emphasis is placed on generality and accuracy, enabling the simulation of diverse fracture phenomena across a range of material systems and loading scenarios.

**Figure 1:**
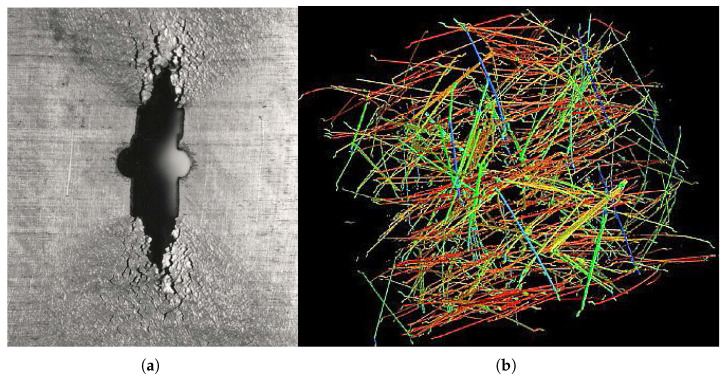
Tested materials: AISI 304L steel and fibre-reinforced cement pasta. (**a**) AISI 304L austenitic steel after creep exposition—crack detail. (**b**) The axonometric projection of separated fibres in a cube specimen with an edge length of 150 mm—CT scan.

Many materials used in engineering applications, such as cement-based composites, which are essential in construction, have complex material structures that make it impossible to accurately predict how they will behave under mechanical, thermal, and other loads using the traditional simplified methods of classical fracture mechanics, as analysed in [2]. A deeper introduction to fracture energy and the effective parameters of concrete can be found in [11]. This served as a compelling incentive for the creation of a more sophisticated method based on cohesive zone modelling (CZM) (see [10]). The aims of physical and mathematical analysis were the following (i) the proper prediction of strains and stresses in both cracked and uncracked structures, including those with different notches; (ii) the admissibility of a non-negligible size of fractured zones in comparison with other dimensions, and (iii) the incorporation of an initial crack in brittle materials, as needed for linear elastic fracture mechanics, the main objectives of the physical and mathematical analysis [12]. The overall framework for interfaces, CZM, is based on the assessment of cohesive forces that arise during the separation of specific material components. The application of CZM to austenitic steel is based on the same approaches. Figure 1 represents both tested materials.

In the following sections, the authors of the article draw on their own experience with programming and creating modifications of the finite element method and its use for modelling structural elements of the above-mentioned materials that they have encountered in recent years. The article also outlines a comprehensive mathematical approach to the model class of static, quasi-static, and dynamic modelling problems. While based on a cohesive approach, some microstructural characteristics of the two materials are not significantly different, since the average grain size of austenitic steel is about 0.1 mm, while the length of the wires in the model cement composite is several millimeters.

## 2. State of the Art and Literature Overview

Despite physical separation, which suppresses any stress singularity and confines it to the cohesive strength of the material, CZM ensures the validity of formal mathematical continuity criteria. However, a specific traction–separation curve that characterizes the constitutive behaviour of a fracture must be identified experimentally for each material separately. In other words, the length of the fracture process zone decreases with the ratio between the maximum stress and the yield stress, while the amount of fracture energy dissipated in the work region depends on the shape of the considered model. CZM can provide accurate predictions for a variety of steels, for various notched samples of a glassy polymer, and even for concrete, as shown by [13,14].

For practical modelling, the Mazars damage model is typically applied (see [15,16] and its regularized version in [17]), mainly to the physically nonlinear analysis of concrete structures, and is based on a strain formulation. The primary goal is to modify the damage model by regularising the evolution rules of both tension and compression damage utilising a traditional fracture energy methodology. Its hypothesis is based on the isotropic behaviour of elastic damage (see [18]). Only one scalar damage variable is defined in this model, which is predicated on the idea that damage only arises from positive strains in the primary directions, which obliquely encourages the emergence of dispersed cracks. The damage model’s isotropic nature, which depicts the material as completely destroyed and limited in time, is the cause of this; no permanent strains are acknowledged, and the unloading path is linear.

Computational fracture mechanics has advanced significantly over the past 20 years. From its original use in purely brittle fracture, cohesive zone modelling (CZM) has developed into complex frameworks that can accommodate ductile and quasi-brittle behaviours. Traction–separation rules enable CZM to describe the fracture process zone. Nonlocal and regularized techniques have been incorporated recently to improve numerical stability and reduce mesh sensitivity. Phase-field models, for instance, provide a diffuse approximation of crack surfaces by regularizing the fracture energy distribution.

## 3. Physical and Mathematical Preliminaries

Interest in the micromechanical models of damage has grown. The main benefit is that, in theory, the parameters of the corresponding models only depend on the material and not the geometry, unlike traditional fracture mechanics. Such concepts ensure transferability over a broad range of sizes and geometries from specimen to construction. The cohesive zone model and the fracture mechanics approach are utilized to predict how cracks would spread through interface parts in the case of an interface element or thanks the XFEM approach within the body; for their potential coupling, cf. [19]. The cohesive model for crack propagation analysis can be integrated into the finite element program using both methods.

A hybrid approach that combines testing and numerical modelling is needed to identify and determine the micromechanical characteristics. An original challenge for micromechanical modelling is that every constituent has a unique microstructure and is not consistent at the microscale. Hill and others first proposed the idea of a representative volume element (RVE). There are several constitutive models for the evolution of damage, such as (i) the nucleation, growth, and coalescence of microvoids (ductile rupture) and (ii) the formation of microcracks and their growth with little global plastic deformation (cleavage fracture)—namely, the node release technique, which can be controlled by any fracture mechanics parameter; the constitutive equation including damage by Gurson, cf. [20,21]; continuum damage concepts based on Kachanov and Lemaitre theory, cf. [22,23]; or the cohesive zone approach following [24], as realized by cohesive elements and/or XFEM. These are some of the methods that can be used to simulate crack propagation within a structure. The most important part of simulating crack growth appears to be modelling and experimentally determining the input parameters for models utilised in FEM (see [25]). Standardizing the modelling methods and the experimental determination of the base data is crucial.

**Figure 2:**
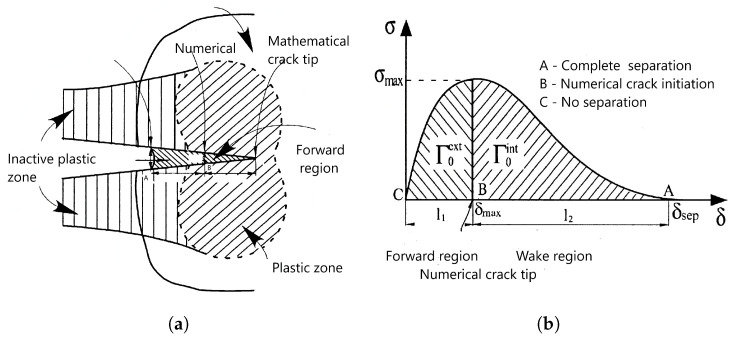
The principle of the cohesive zone approach in conjunction with the exponential traction separation law suitable for the initial plastic area. $\Gamma_{0}^{\mathrm{ext}}$ represents the energy in the forward region, which is needed to start crack growth, and $\Gamma_{0}^{\mathrm{int}}$ represents the energy in the wake region, where the crack grows. (**a**) The principle of the cohesive zone approach. (**b**) Traction separation law.

Since the cohesive model is a phenomenological model, there is no evidence of which form to take for the cohesive law. Thus, cohesive law has to be assumed independently of the specific material as a model of the separation process. Most authors adopt their own formulation for the dependence of traction on separation. The cohesive laws presented in Figure 2 schematically show the relation between CZM and traction separation law, but more information can be found in [26]. Detailed procedures for obtaining the traction separation law form for fibre composites (also focusing on the polymer matrix) were approved by ASTM for both fundamental modes (I and II) in D5528/D5528M-21 [27] and D7905/D7905M-19e1 [28]. This issue is carefully elaborated upon in practical examples for mode II in [29], including the effect of the size of the test specimen and the transfer of this data to the real size. Rotationally symmetric bodies, digital image correlation, and supporting deformation analysis are used. Validation analysis is carried out for different loading levels on a statistically significant number of bodies. The total deformation energy is obtained using standard fracture mechanics procedures; then, a simple model of the traction separation law is obtained, which is further verified.

A hybrid technique has been devised to determine the cohesive stress in the situation of normal fracture. The distribution of the axial stress over the specimen geometry’s notch section at the moment of crack initiation in the specimen’s centre is ascertained using standard elastic–plastic analysis. In that case, the axial stress reaches its maximum in the specimen’s centre, which should be equal to $T_{0}$. Assuming that $\Gamma_{0}$ equals the *J* integral at the start of stable crack extension ($J_{i}$), one can calculate the cohesive energy ($\Gamma_{0}$) in a fracture mechanics test.

**Figure 3:**
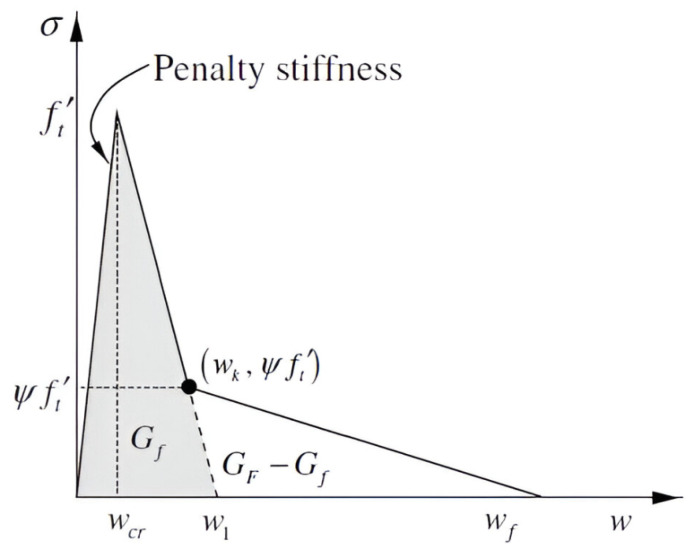
Traction separation law for cement and cement composites reinforced by steel fibres. $w_{1}$ is theoretical displacement for pure cement, $w_{cr}$ is displacement in the forward region, $w_{f}$ is final displacement, $G_{f}$ represents the energy for pure cement, $G_{F}$ is complete deformation energy, $G_{F}-G_{f}$ represents the energy for the bridging effect, and $\psi f_{t}^{\prime }$ is the position on the traction separation curve where simple fibre pulling ends and the bridging effect begins.

While the traction separation law for austenitic steel corresponds to Figure 2, for cement fibre composites, Figure 3 is more descriptive. Crack propagation is controlled according to the principles of fracture mechanics, where the curve integral determining the deformation energy plays a major role. In both linear and nonlinear fracture mechanics, curve integrals are crucial. The most well-known is the *J* integral, whose concept is precisely described in [30,31]; refer to [32,33,34] for the equation’s basic plastic damage. Ref. [35] first defined the C integral, a comparable integral for bodies exposed to creep deformation; see [36,37] for the development of its theory and applications.

**Figure 4:**
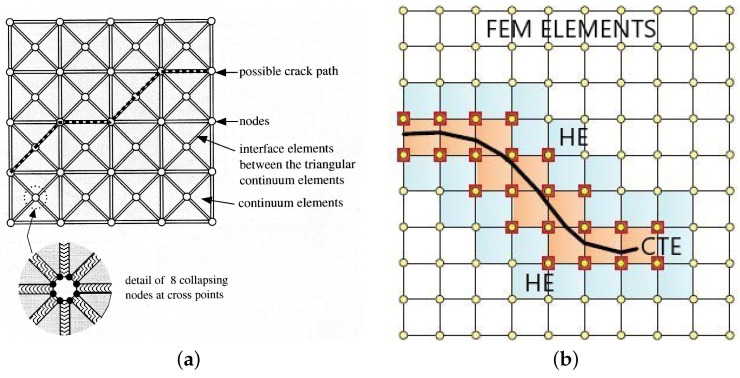
Schematic diagram comparing the principle of crack growth modelling for cohesive elements and elements for XFEM. (**a**) Use of cohesive elements, standard elements, and thin cohesive elements. (**b**) Use of linear XFEM elements (coloured); CTE indicates crack-tip enrichment, and HE represents Heaviside enrichment.

Although XFEM is preferably used in numerical analysis of boundary value problems for differential equations, as this article has already mentioned, the XFEM mesh may not always be the best choice for simulating crack propagation. A similar method based on the contact problem is the use of cohesive elements, which are inserted into the areas where crack growth is expected. Their thickness is actually zero; the problem is that the crack runs only through these cohesive elements, as can be seen in Figure 4. This is among the most significant areas of interest in solid-body mechanics difficulties. Using the variational concept, the initial models were developed on a weak (deformation) discontinuity that could flow through the finite element mesh [38]. By altering the virtual work principle (which also applies to models with the traction separation law), as investigated in [39,40], adding the stability and convergence of such problems and increasing the modelling accuracy. Other authors and researchers have regarded it as a strong (displacement) discontinuity.

In [41,42], the process for applying XFEM to initial and boundary-value time-dependent problems is described in full. Numerous variations have been made to this approach, some of which are even known by different names. Recent advancements can be observed by comparing the work reported in [43] with adjustments made in [44] or in [45] using the boundary element method. In [46], a process for randomly oriented fibres is described; the case of reinforced concrete provides an illustration of a true distribution. However, when the non-local Eringen model [47] modifies the stress field, the models explaining the production of microcracks [48,49] produce different results. A comparison of intrinsic and extrinsic modifications of XFEM can be found in [50,51]; the research progress in the development of XFEM algorithms in the last decade is reviewed in [52,53].

Notice that some recent promising approaches incorporating CZM cannot be easily classified as upgrades of XFEM (or some similar modification of FEM). For example, [54] implemented an arbitrary Lagrangian–Eulerian (ALE) approach to align the crack path with the direction of its propagation obtained from a local stress criterion. Unlike this, ref. [55] replaced FEM (and its extensions) with the element-free Galerkin method (EFG), inspired by the interpolating moving least squares approach (MLS) proposed in [56,57]. Both [54,55] explore 2D elastic formulations supplied by CZM on interior interfaces; therefore, further generalizations would be welcomed.

Another nontrivial set of research motivations can be seen in the effective integration of all classical loading modes (relative to the crack: I, opening; II, shearing; III, tearing) into a general mixed-mode computational tool, as demonstrated by [58] for the case of 3D linear fracture mechanics. Although the examples presented in this article are related to mode I for simplicity, such computations can be covered by XFEM techniques, although the reliability of their results strongly depends of the setting of *K* factors based on well-designed experiments; for two special Al-based alloys, such an experimental study is presented in [59]. For arbitrary 3D interacting crack networks, a regularized XFEM (RXFEM) technique supplied by CZM with regularized Heaviside functions on all interfaces was developed in [60].

The usual approach to mathematical modelling, incorporating the phenomena of crack initiation and development introduced above, relies on the conservation principle of classical thermomechanics, as discussed in [61]—namely, the conservation of mass, (linear and angular) momentum, and energy. For the sake of simplicity, in the following formulae, we attempt to get by on the weak formulation of energy and a small strain simplification for a quasi-static case supplied by special, semi-empirical constitutive equations; selected generalizations will be referenced later. The constitutive equations presented here rely on the so-called SLS (standard linear solid) viscoelastic simplification, introduced in [62].

In general, a deformable body (Ω) is allowed to occupy a domain in the three-dimensional Euclidean space ($R^{3}$), which consists of a finite number (*n*) of disjoint parts ($\Omega_{i}$) with i∈{1,…,n} and Lipschitzian boundaries $\Lambda_{ij}$ between $\Omega_{i}$ and $\Omega_{j}$, where i,j∈{1,…,n}, j≠i (some of them may be empty). From such sets, both the exterior boundary (∂Ω) of Ω and the set of all interior interfaces (Λ) can be created, the properties of which should be introduced separately. Standard boundary conditions can be implemented on ∂Ω, whose two disjoint parts can be distinguished as Θ with Dirichlet boundary conditions (whose Hausdorf measure on ∂Ω must be positive) and Γ with Neumann boundary conditions.

A Cartesian coordinate system ($x=(x_{1},x_{2},x_{3})$) can be implemented in $R^{3}$; then, the displacement ($u\left(x,t\right)=(u_{1}\left(x,t\right),u_{2}\left(x,t\right),u_{3}\left(x,t\right))$) of any point of Ω, Θ, Γ, and Λ, dependent on time *t*, can be related to its initial state with t=0. Tor a time interval (I=[0,T]) of a finite length (T), we are able to set the Cauchy initial condition to u(.,0)=o on Ω, where o=(0,0,0).

Weak formulations of problems of computational mechanics need the notations and results from the theory of (abstract) function spaces, namely the Lebesgue, Sobolev, and Bochner–Sobolev spaces here, we shall work with those from [63]. Namely, the following notations of such spaces will be needed:

$$H=L^{2}{\left(\Omega \right)}^{3}\;,\;\;\;E=L^{2}{\left(\Omega \right)}_{\mathrm{sym}}^{3\times 3}\;,\;\;\;G=L^{2}{\left(\Gamma \right)}^{3}\;,\;\;\;G=L^{2}{\left(\Lambda \right)}^{3}\;,$$

$$Z=L^{\infty }\left(\Omega \right)\;,\;\;\;M=L^{2}{\left(\Omega \right)}_{\mathrm{sym}}^{\;(3\times 3)\times (3\times 3)}\;,\;\;\;V=\left\{v\in W^{1,2}{\left(\Omega \right)}^{3}:\;v=o\;\mathrm{on}\;\Theta \right\}\;,$$

together with scalar products in special Hilbert spaces ((.,.) in H, 〈.,.〉 in G ${\langle .\;,.\rangle }_{*}$ in G and ((.,.)) in E), i.e.,

$$\begin{array}{ccc} \left(\tilde{\varphi },\varphi \right)=\sum_{i=1}^{3}\int_{\Omega }{\tilde{\varphi }}_{i}\left(x\right)\varphi_{i}\left(x\right)\;dx\;\;\;\; & & \;\;\;\;\;\;\mathrm{for}\;\mathrm{all}\;\tilde{\varphi },\varphi \in H\;,\\ \left(\;\left(\tilde{\varphi },\varphi \right)\;\right)=\sum_{i,j=1}^{3}\int_{\Omega }{\tilde{\varphi }}_{ij}\left(x\right)\varphi_{ij}\left(x\right)\;dx & & \;\;\;\;\;\;\mathrm{for}\;\mathrm{all}\;\tilde{\varphi },\varphi \in E\;,\\ \langle \tilde{\varphi },\varphi \rangle =\sum_{i=1}^{3}\int_{\Gamma }{\tilde{\varphi }}_{i}\left(x\right)\varphi_{i}\left(x\right)\;\mathrm{ds}\left(x\right) & & \;\;\;\;\;\;\mathrm{for}\;\mathrm{all}\;\tilde{\varphi },\varphi \in G\;,\\ {\langle \tilde{\varphi },\varphi \rangle }_{*}=\sum_{i=1}^{3}\int_{\Lambda }{\tilde{\varphi }}_{i}\left(x\right)\varphi_{i}\left(x\right)\;\mathrm{ds}\left(x\right) & & \;\;\;\;\;\;\mathrm{for}\;\mathrm{all}\;\tilde{\varphi },\varphi \in G\;. \end{array}$$

Notice that the same products can be considered as dualities between special Banach spaces and their adjoint spaces. In particular, instead of H, with $\tilde{\varphi }\in L^{\tilde{p}}{\left(\Omega \right)}^{3}$ and $\varphi \in L^{p}{\left(\Omega \right)}^{3}$, any real p>1 and $\tilde{p}=p/\left(p-1\right)$ in the first equation; this corresponds to a Hilbert space just for $p=\tilde{p}=2$. However, we shall avoid such a generalization now for simplicity.

For a fixed t∈I and an arbitrary γ∈E, [((γ,τ))] must be understood in the sense of the Bochner integral of an abstract function $\left(\;\left((\gamma ,\tau \left(.\;,\tilde{t}\right)\right)\;\right)$ over $\tilde{t}\in \left[0,t\right]$ where $\tau \in L^{2}\left(I,E\right)$; see [63], Part 7.1). We consider a linear strain tensor (ε(v)∈E) with the following components:

(1) $$\varepsilon_{ij}\left(v\right)=\left(\partial v_{i}/\partial x_{j}+\partial v_{j}/\partial x_{i}\right)/2$$

where i,j∈{1,2,3} can be defined for any virtual displacement (v∈V).

## 4. A Model Problem

Using the notations introduced aboved, the conservation of momentum, in its weak form, reads as follows for an arbitrary v∈V and any t∈I:

(2) $$\left(\;\left(\varepsilon \left(v\right),\sigma \right)\;\right)+{\langle ↕\;\;v\;\;↕,\varsigma \rangle }_{*}=\left(v,f\right)+\langle v,g\rangle$$

**Figure 5:**
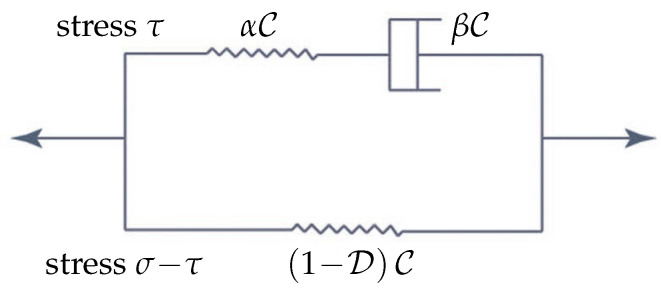
Scheme of the viscoelastic standard linear solid (SLS) model with damage.

The constitutive equations (i) between the total stress ($\sigma \in L^{2}\left(I,E\right)$) and the strain (ε(u)) on Ω and (ii) of the interior surface traction ($\varsigma \in L^{2}\left(I,G\right)$) and the interface jumps (↕u↕ of *u*, in the sense of traces) must be pre-defined on all parts of Λ. The evaluation of ς=T(.,↕u↕) for a function (T) measurable in its first argument on Ω (to handle inhomogeneous materials) and continuous in its second argument from ($R^{3}$) (usually corresponding to ↕u↕ with *u* decomposed to its 1 normal component and 2 tangential components) may be rather complicated; see the discussion on traction–separation laws above. This can be inserted directly into (2). Unlike this, the evaluation of σ must take both branches of the scheme presented in Figure 5, corresponding to the SLS model, into consideration.

In Figure 5 C∈M is the stiffness tensor, α and β∈Z are certain scalar material factors, and D∈Z is considered as a damage factor whose increase forces the loss of stiffness. It is worth noting that in the case of an isotropic material C can be composed only of two independent characteristics–namely, Young’s modulus (*E*) and the Poisson’s ratio (ν). It is reasonable to suppose that the existence of such a positive constant *c* where max(α(x),β(x))≥c holds for any x∈Ω yields together with $a^{T}C\left(x\right)a\geq ca^{T}a$ for an arbitrary matrix $a\in R_{\mathrm{sym}}^{3\times 3}$.

**Figure 6:**
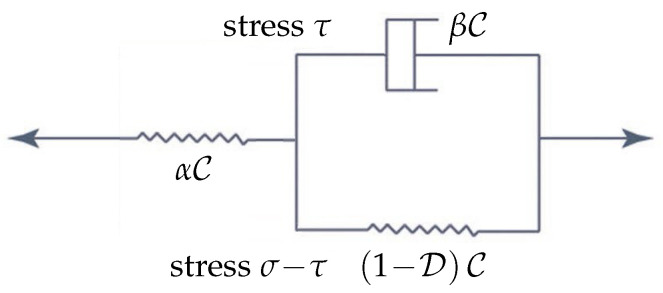
Scheme of the simplest viscoelastic Kelvin chain (KCH) with damage.

An alternative to SLS, frequently referred to as the Kelvin chain (KCH), is demonstrated in Figure 6, where only the simplest KCH is shown; more component Kelvin chains can be derived by concatenation of the classical Kelvin scheme shown on the right-hand side of Figure 6 (with different material parameters in general) [64], with the incorporation of damage suggested in [65]. In the classical nomenclature, with zero-valued D, Figure 5 represents the Zener model, and Figure 6 shows the Boltzmann model; for an overview of further composed models (Tsay, Burgers, Weichert, etc.) not covered by KCHs directly, see [66]. However, we shall work with SLS in most of the following considerations for simplicity.

The evaluation of σ must take both branches of the scheme shown in Figure 5 into consideration. The upper branch (σ=τ+(1−D(u))Cε(u)) takes the form of an additional integral equation with an unknown τ, i.e.,

(3) $$\left(\;\left(\gamma ,C^{-1}\tau /\alpha \right)\;\right)+\left[\left(\;\left(\gamma ,C^{-1}\tau /\beta \right)\;\right)\right]=\left(\;\left(\gamma ,\varepsilon \left(u\right)\right)\;\right)\;,$$

whereas (2) takes the form of

(4) $$\left(\;\left(\varepsilon \left(v\right),\tau \right)\;\right)+\left(\;\left(\varepsilon \left(v\right),\left(1-D\left(u_{s}^{m}\right)\right)\;C\varepsilon \left(u\right)\right)\;\right)+{\langle ↕\;\;v\;\;↕,T(↕\;\;u\;\;↕)\rangle }_{*}=\left(v,f\right)+\langle v,g\rangle \;.$$

It is reasonable to assume $f\in L^{2}\left(I,H\right)$ for volume loads and $g\in W^{1,2}\left(I,G\right)$ for surface loads in (4). Then, a model problem can be read as follows: find $u\in L^{2}\left(I,V\right)$ and $\tau \in L^{2}\left(I,E\right)$ such that (3) and (4) hold for any t∈I; two initial conditions of u=o and τ=O are considered, with *O* being the zero matrix from $R^{3\times 3}$.

Unlike T(.), the evaluation of a bounded continuous function (D(.)), interpreted as a damage factor, respecting the irreversibility of damage, needs some regularisation. The proper mathematical discussion of such a five-step algorithm, making use of the intuitive nonlocal Eringen approach [47] based on the regularisation kernels from $L^{2}\left(\Omega \times \Omega \right)$ proposed in [67] was performed in [68]. In general, we need $D(.,\tilde{\sigma })$ as a function measurable in its first argument on Ω and continuous in its second argument from $R^{3}$, where $\tilde{\sigma }\in H$, as derived from *u*, is a certain regularised vector of stress-invariants. The existence of a solution based on the method of discretisation in time and the properties of Rote sequences was verified for a standard Kelvin quasi-static viscoelastic model (i.e., that corresponding to Figure 6 without the left-hand-side elastic component) in [69] and for an analogous dynamic component in [70]. Similar results (after rather long computations) can be derived for the (3) and (4) system of evolution by combining the approaches proposed in [69,70] with that described in [62].

Notice that such an approach admits natural generalizations in several directions. Namely, as shown in Figure 6, KCH can work with σ=C(ε(u)−η) in (4), with (3) replaced by

[((γ,(1+α)Cη))]+((γ,βCη))=[((γ,(1−D(u))Cε(u)))].

Thus, we have to seek $u\in L^{2}\left(I,V\right)$, accompanied by $\eta \in L^{2}\left(I,E\right)$, instead of $\tau \in L^{2}\left(I,E\right)$. For more details on generalized KCHs, their possible differential and integral formulations, and related computational algorithms, see [64,65] for deep mathematical results on a still wider class of such models (not limited to viscoelasticity) (cf. [71]).

Still, other partial generalisations may be required because of the nonlinearity of some symbolic components contained in Figure 5 or Figure 6; namely, the use of Norton power-law creep as a viscous component brings an additional nonlinear term, which must be handled properly (see [72]). Unlike all evolutionary approaches, some computational models try to cover irreversible deformation by the implementation of special yield surfaces inside the usual elastoplastic theory, e.g., for cementitious composites, the Rankine–Hill theory, following [73,74]. A research direction based on the implementation of fractional derivatives in constitutive equations is documented in [75].

Nevertheless, numerous substantial generalisation are needed in practical engineering computations, although their transparent mathematical analysis as quasilinear problems in Hilbert or sufficiently simple reflexive Banach spaces is still not available. Some examples follow:

(i) Removal of the linearised strain assumption, characterised by (1), can be handled using a geometry update like that described in [76] or by applying the ALE formulation in a similar way to that reported in [54]. However, such an approach may not guarantee the preservation of all assumptions on Ω compounded from subdomains with non-Lipschitzian boundaries; for such domains, only much weaker results are available in the advanced functional analysis, cf. [77,78].
(ii) The proper physical analysis, following [14], avoiding (semi-)empiric relations, should expresses the specific Gibbs energy as a function of four state variables, namely temperature, stress, damage, and a set of internal parameters corresponding to four dissipative variables involved in a dissipative potential whose partial differentiation works with the theory of subdifferentials.
(iii) Special physical considerations are needed in the case of fast contacts/impacts of deformable bodies, with accent on the detailed analysis of dissipative energy during such process, cf. [79]. In the case of contacts of multiple bodies [80], non-trivial results and algorithms from graph theory for the effective search for contact candidates cannot be avoided.
(iv) Moreover, the finite strain approach switches such considerations to the (still more complicated) theory of structured deformation (see [81], Chapters 8 and 9, and [82]). Thus, up to now, all computational approaches have been subject to certain compromises between the complexity of physical and mathematical formulations and the need for inexpensive, effective, and robust computational tools.

## 5. Computational Issues

Let I be covered by a finite number (*m*) of subintervals ($I_{s}^{m}$), introduced as the sets of all *t* satisfying (s−1)h<t≤sh, where s∈{1,…,m}. The notation of h=T/m will be used for brevity. Then, we can rewrite (3) and (4) in their time-discretised forms:

(5) $$\left(\;\left(\varepsilon \left(v\right),\tau_{s}^{m}\right)\;\right)+\left(\;\left(\varepsilon \left(v\right),\left(1-D\left(u_{s}^{m}\right)\right)\;C\varepsilon \left(u_{s}^{m}\right)\right)\;\right)+\langle ↕\;\;v\;\;↕,T(↕\;\;u_{s}^{m}{\;\;↕)\rangle }_{*}=\left(v,f_{s}^{m}\right)+\langle v,g_{s}^{m}\rangle \;,$$

(6) $$\left(\;\left(\gamma ,C^{-1}\left(\tau_{s}^{m}-\tau_{s-1}^{m}\right)/\alpha \right)\;\right)+h\left(\;\left(\gamma ,C^{-1}\tau_{s}^{m}/\beta \right)\;\right)=\left(\;\left(\gamma ,\varepsilon \left(u_{s}^{m}-u_{s-1}^{m}\right)\right)\;\right)\;.$$

Clearly, the analysis of limit passage m→∞ (or $h\to 0_{+}$) is required. Unlike $f_{s}^{m}$ and $g_{s}^{m}$, which can be taken as appropriate approximations at the required precision level, $u_{s}^{m}$ and $\tau_{s}^{m}$ are unknown in advance.

Since (5) and (6) are still in infinite-dimensional function spaces, some discretisation on Ω and all interfaces is needed for practical calculations, typically that forced by XFEM. This can be done using the following formulae:

(7) $$\left(\;\left(\varepsilon \left(v^{\delta }\right),\tau_{s}^{m}\right)\;\right)+\left(\;\left(\varepsilon \left(v^{\delta }\right),\left(1-D\left(u_{s*}^{m\delta }\right)\right)\;C\varepsilon \left(u_{s}^{m\delta }\right)\right)\;\right)+\langle ↕\;\;v^{\delta }\;\;↕,T(↕\;\;u_{s*}^{m\delta }{\;\;↕)\rangle }_{*}\;=\left(v^{\delta },f_{s}^{m}\right)+\langle v^{\delta },g_{s}^{m}\rangle$$

for all $v^{\delta }\in V^{\delta }$ and

(8) $$\left(\;\left(\gamma^{\delta },C^{-1}\left(\tau_{s}^{m\delta }-\tau_{s-1}^{m\delta }\right)/\alpha \right)\;\right)+h\left(\;\left(\gamma^{\delta },C^{-1}\tau_{s}^{m\delta }/\beta \right)\;\right)=\left(\;\left(\gamma^{\delta },\varepsilon \left(u_{s}^{m\delta }-u_{s-1}^{m\delta }\right)\right)\;\right)\;.$$

for all $\gamma^{\delta }\in G^{\delta }$, with δ understood as some reference mesh-element edge length. $G^{\delta }$ and $V^{\delta }$ in (7) and (8) are finite-dimensional approximations of G and V (or just their subspaces in some simple particular cases). In other words, we come to a system of a finite number of (in general, nonlinear) algebraic equations whose effective solution is required; the convergence of such a method depends on the behaviour of (7) and (8) during the limit passage (δ→0). Namely, $u_{s*}^{m\delta }$ is a certain step-by-step approximation of $u_{s*}^{m\delta }$ for s∈{1,…,m}; e.g., the choice of $u_{s-1}^{m\delta }$ can be seen as a starting point of an appropriate iterative procedure inside a time step (*s*). Numerical methods such as the inexact Newton method, the nonlinear conjugate gradient method, and the Nelder–Mead simplex method are available. A preference for just one such method is not possible; this depends of different techniques of formulation and evaluation of T(.) and D(.). As a nontrivial example, ref. [83] tries to overcome the difficulties with multiple contacts using the so-called semi-FEM, implementing the bipotential theory and combining the Newton and Uzawa iterative processes.

## 6. Illustrative Examples

The first experimental material is austenitic steel, where fracture behaviour can be modelled using an exponential traction–separation law. The actual experiments were performed on steel designated as AISI 304L during the first author’s stay at the Institute of Physics of Materials of the Czech Academy of Sciences. Flat samples of 72 × 30 × 10 mm were made from the the above-mentioned material. The a priori crack was drilled, and starting grooves were created by electrospark discharge. The central crack was created by transient loading, and standard cylindrical bodies were used to obtain creep data. The nature of long-term tests does not allow for easy identification of the traction–separation law, a simple exponential form proven to be suitable for numerical modelling. The critical value of $J^{˙}$ (or $C^{*}$) was taken from [36]. However, both integrals are identical for stationary creep in the case where a zero stress rate leads to a zero elastic strain rate, and this value is denoted $C^{★}$, which leads to the result of $\lim_{t\to \infty }C\left(t\right)=C^{★}=J^{˙}$, as considered in [84].

**Figure 7:**
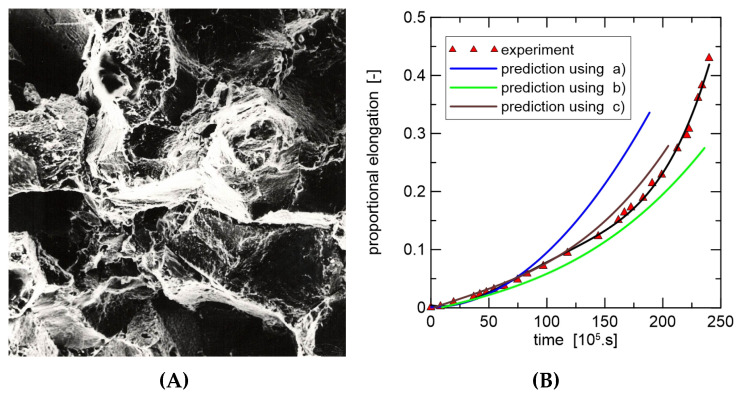
Crack growth detail of optical microscopy and numerical modelling; stress on the residual cross-section is 50 MPa. (**A**) Portion of cavitation damage of grain boundaries; magnification: 180×. (**B**) Experiment versus modelling using XFEM; $J^{˙}$ = (a) 40, (b) 45, (c) 50 N/mm/h.

**Figure 8:**
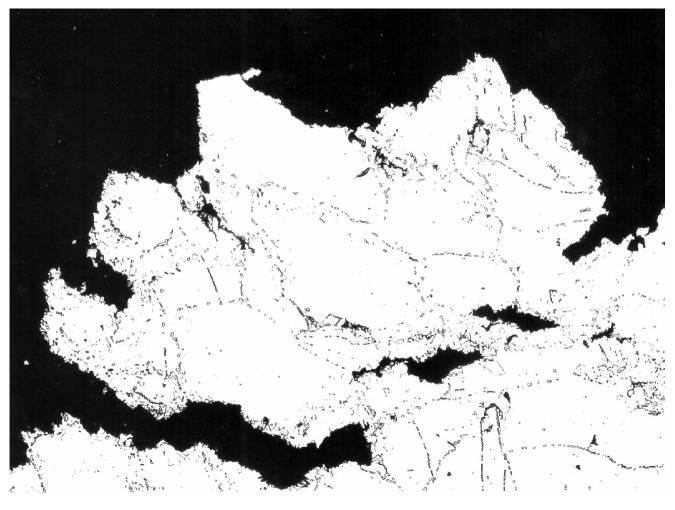
The initiation of a secondary crack with a pronounced intercrystalline character of failure, which, in the final state of damage, becomes transcrystalline; magnification: 100×.

When using XFEM, the initial task is to idealise the physical phenomenon using a mathematical model rather than to construct the finite element mesh. The high rate of the intercrystalline violation over almost the whole creep exposure, as demonstrated in the preceding sections and experimental data, makes it easier to develop the model and apply the criterion for crack propagation. Table 1 provides a summary of the input material properties used for crack propagation modelling. The problem was resolved like a flat strain because of the geometry of the bodies that were studied (see Figure 7). For the stress study, two modifications were made to the FEM mesh (1000 and 10,000 2D elements). Figure 8 informs us about what the microstructure looks like in the final part of the damage.

**Figure 9:**
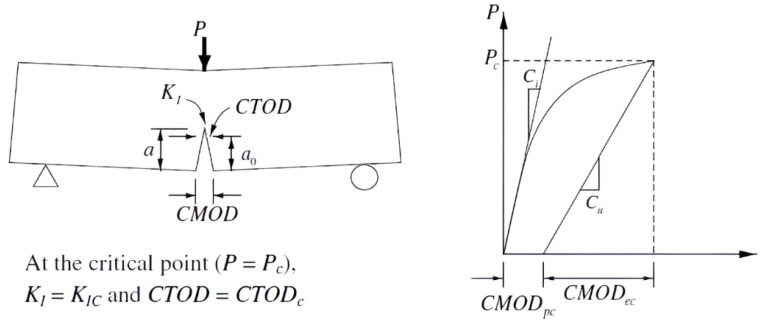
Schematic diagram of the concrete test.

**Figure 10:**
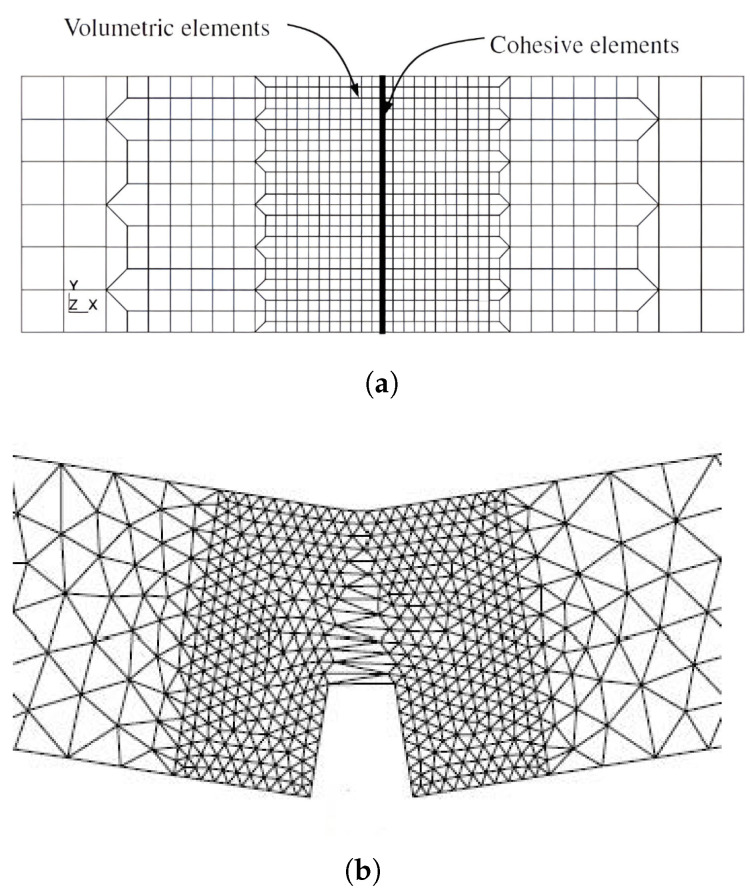
Crack growth modelling using cohesive elements. Some elements are opened, and in the rest of the elements, one can see the shift in the traction–separation law. (**a**) Initial mesh used for cohesive modelling via cohesive elements. (**b**) Crack opening and the end of crack growth.

**Figure 11:**
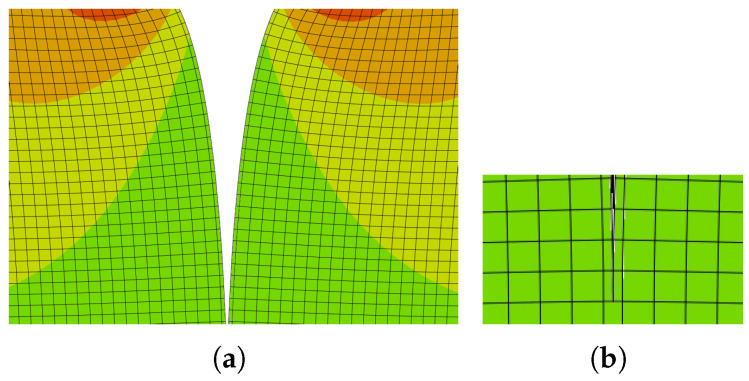
The path is perpendicular to the linear XFEM element in the central part of the linear element of initial crack growth. (**a**) Crack growth modelling using linear XFEM elements. (**b**) Detail of the crack tip.

As shown in Figure 9, a sample containing steel fibres and a cement matrix was chosen for computational modelling. Numerical results demonstrate how the location and material parameters affect the surface propagation of cracks in the injured body. The direction of crack propagation is significantly influenced by the reinforcing effect of the bridging. With a parabolic partial system differential equation, special attention is given to the Eringen model for the generation of the multiplicative damage factor, associated quasi-static analysis, the existence of a weak solution of the relevant boundary value, and the initial value problem. Thus, the suggested process integrates the potential of multiple methods for simulating the spread of cracks in fibre composites. This problem was modelled using both cohesive elements Figure 10 and the XFEM method( Figure 11), but the same traction–separation law was used in both cases.

**Figure 12:**
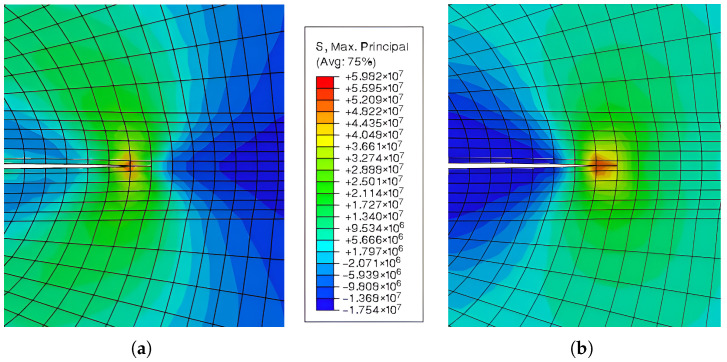
Crack growth modelling using XFEM. The critical stress intensity factor ($K_{IC}$) is 0.5 ${\mathrm{MPa.m}}^{1/2}$. (**a**) Crack growth modelling via the Mazars model. (**b**) Crack growth modelling via the Mazars model with Eringen regularization.

As an example, a reasonably simple two-dimensional body that satisfies the planar strain criterion was selected. The following cracks are assumed to form from this stress concentrator utilising modified XFEM, since a uniform load was applied to the surface of this a priori fracture. In order to model matrix damage based on planar element CPE4, a user subroutine in the Fortran 90 language was implemented in commercial Abaqus 2018 software, which served as the foundation for the calculation system representing the bridging effect, corresponding to Figure 12. Young’s modulus (E=3.2 GPa), Poisson’s ratio (ν=0.3), and the tensile strength (10 MPa) were the fundamental input data used for this task, corresponding to reinforced cement paste. These data were used based on measurement results for various model materials measured at the Faculty of Civil Engineering and materials presented in the literature (e.g., [85]); the compressive strength was measured, but the tensile strength was estimated as 8 percent of this value. In ultra-high-toughness cementitious composites, the volume of fibres is nearly 4 percent, and the fibre length is about 30 mm.

The results of the well-established Mazars model are presented in Figure 12 (left figure). One example of a pure smeared Eringen model is shown in the same figure on the right. The Mazars damage model is typically applied to the physically nonlinear analysis of concrete structures and is based on a strain formulation. The primary goal is to modify the damage model by regularising the evolution rules of both tension and compression damage utilising a traditional fracture energy methodology. Its hypothesis is based on the isotropic behaviour of elastic damage. Indirectly encouraging the emergence of smeared cracks, this concept is predicated on the idea that damage can only arise from positive strains in the primary directions. Because the damage model is isotropic, there is just one scalar damage variable defined, which depicts the material as completely damaged and has a limited interval. Since the unloading path is linear and no permanent strains are introduced, no hysteresis loops are displayed.

## 7. Results and Discussion

The findings on austenitic steel confirmed that dislocation processes are significant, fracture can be described by power-law creep, and time to fracture is influenced by stress. The scenario that was chosen points to an initial state reaction that is completely elastic. Therefore, stress relaxation causes a progressive transition from exclusively intergranular fracture to mixed and ultimately transgranular failure. The overall modelling optically provides a more precise match with the experiment, and the real value of the $J^{˙}$ integral varies only in the final minutes of creep damage. The use of the XFEM, as well as cohesive elements with the exponential traction–separation law, seems to be a convenient approach for creep growth modelling. It should be noted that for this type of material, we would obtain very similar results using cohesive elements; the exponential form of the separation law as the bridging effect is significantly less pronounced than in structural fibre composites. In this case, we obtain good agreement when modelling the propagation of a main crack, but in the case of crack branching, the use of cohesive elements is questionable. The critical value of the $J^{˙}$ integral is assumed to be constant, but it is logical that with a change in the failure mechanism in the last tenth of the service life, this value will also change slightly.

A cement matrix reinforced with metal fibres was used for the ensuing numerical testing. The most notable example is a cement composite with short steel fibres that are intentionally or very randomly oriented, often combined with ceramic or polymer fibres that primarily lower specific stress components. A more comprehensive mathematical description is expected. The significance of the averaged stress distribution before the fracture tip is also covered, even though we have already touched on a number of techniques for evaluating damage in these composite materials and structures. Based on common laboratory tests and observations, new trends in numerical modelling and simulation are required that take into account the advancement of sophisticated materials, structures, and technologies, as well as the extensive expertise of their designers. In some complex scenarios, the stability of the XFEM discretisations is straightforward for open cracks when crack-tip effects are ignored, but it becomes difficult if we require the crack to be closed. Fibre-reinforced composites have had to replace metal alloys due to the aerospace, automotive, and marine industries’ need for lightweight, high-strength materials.

Experimental works in setting of CZMs, namely the design of traction–separation laws, are crucial for reasonable numerical modelling and simulation of both laboratory testing (under intentionally simplified geometrical and physical aonditions) and real behaviour of advanced material samples and structures; this holds in a much more general context than for the two examples presented in the previous section. The appropriate form of such work is material-dependent, with such considerations documented, e.g., (i) for composite adhesive joints with nanoparticles in fracture modes I and II in [86], cf. [87] for crack propagation mechanisms in sintered copper nanoparticles, too; (ii) for interlaminar damage in composite laminates in [88]; or (iii) for laminated glass, consisting of a polymeric interlayer such as PVB (polyvinyl butyral) sandwiched between two glass plies in [89]. Attention must also be paid to the size of the elements used in front of the crack front, i.e., their size should reflect the physical nature of the dominant processes controlling damage and crack growth (sometimes the term representative volume element is used). The size of the elements for the finite element method and its modifications must be significantly smaller.

From the computational point of view, some reasonable XFEM convergence estimates are needed as a support for the design of effective algorithms. Experimental convergence investigations of XFEM are documented in [42]. Theoretical estimates of the convergence rate, working with δ (and its powers) from (7) and (8), or some comparable equations in appropriate Sobolev spaces, which are common in the standard FEM, can be derived for a sufficiently simple system of cracks, as for one straight crack for the modelling of problem in [90]. Another result for the error estimates with $\delta^{p}∥.∥$ and 1<p≤4 in certain $L^{2}$- and energy norms ∥.∥, referring to a model Poisson problem in $R^{2}$ with both straight and quadratically curved discontinuities, can be found in [91]. A machine learning-supported generalized FEM for implementation in interface problems was recently suggested in [92]. However, optimal design of XFEM basis functions for crack systems in real engineering problems based on weak formulations of initial and boundary-value problems for partial differential equations of evolution is expected to remain a research challenge in the coming yearsB.

## 8. Conclusions

The main results can be characterized as follows:

- The suitability of the modified finite element method for two different types of materials was demonstrated. Several types of smeared models were tested as part of the modifications of this method.
- New trends in the modification of the classical finite element method with more sensitive modelling of the influence of the microstructure were indicated and commented on.
- A brief analysis of new procedures based on recommendations and standards in recent years for determining the traction–separation law was performed. The presented examples based on original calculations and experiments prefer loading in mode I.
- In the theoretical part, a mathematical analysis of the modified finite element method for static, quasi-static, and viscous deformation of loaded bodies was performed.
- In the case of austenitic steel, of course, several mesh variants were tested. The more significant influence was the setting of the critical value of the $C^{★}$ integral. The results obtained using the XFEM and cohesive elements were similar. The task is much more sensitive to the choice of the criterion governing crack formation.
- The cement composite used for the presented results was a model material, and the onset of the bridging effect was estimated using data typical for a class of similar materials.

Research challenges can be seen both in the design of optimal XFEM-based algorithms for arbitrary loading modes and in the mathematical verification of the solvability of problems generated by (3), (4), etc., together with related experimental validation. This generates an additional set of inverse problems of reasonable setting of material parameters for computational modelling.

## Figures and Tables

**Table 1:** Applied values of input material parameters.

Input Data		Value
*E*	Young modulus	$1.45\times 10^{5}$ MPa
ν	Poisson ratio	0.3
$\sigma_{2}$	Yield strength	145 MPa
$E^{\prime }$	Slope of the plastic part of the true stress × true strain curve	700 MPa
*B*	Creep proportionality constant for power law	$1.36\times 10^{-20}$ ${\mathrm{MPa}}^{-7}$ $s^{-1}$
*n*	Creep stress sensitivity parameter	7

## Data Availability

The original contributions presented in this study are included in the article. Further inquiries can be directed to the corresponding author.
